# Protection Against Transplacental Transmission of a Highly Virulent Classical Swine Fever Virus Two Weeks After Single-Dose FlagT4G Vaccination in Pregnant Sows

**DOI:** 10.3390/vaccines13080803

**Published:** 2025-07-28

**Authors:** Liani Coronado, Àlex Cobos, Adriana Muñoz-Aguilera, Sara Puente-Marin, Gemma Guevara, Cristina Riquelme, Saray Heredia, Manuel V. Borca, Llilianne Ganges

**Affiliations:** 1 WOAH Reference Laboratory for Classical Swine Fever, IRTA-CReSA, 08193 Barcelona, Spain; liani.coronado@irta.cat (L.C.); adriana.munoz@irta.cat (A.M.-A.); sara.puente@irta.cat (S.P.-M.); cristina.riquelme@irta.cat (C.R.); saray.heredia@irta.cat (S.H.); 2 Unitat Mixta d’Investigació IRTA-UAB en Sanitat Animal, Centre de Recerca en Sanitat Animal (CReSA), Bellaterra, 08193 Barcelona, Spain; alex.cobos@irta.cat (À.C.); gemma.guevara@irta.cat (G.G.); 3 IRTA, Programa de Sanitat Animal, Centre de Recerca en Sanitat Animal (CReSA), Bellaterra, 08193 Barcelona, Spain; 4 Instituto Colombiano Agropecuario (ICA) Subgerencia de Análisis y Diagnóstico, Bogotá 110911, Colombia; 5 Plum Island Animal Disease Center, Agricultural Research Service, United States Department of Agriculture, Greenport, NY 11944, USA

**Keywords:** classical swine fever virus, transplacental transmission, FlagT4G vaccine, early protection, immune response, vaccine efficacy, pregnant sows

## Abstract

Background/Objectives: Classical swine fever (CSF) continues to challenge global eradication efforts, particularly in endemic regions, where pregnant sows face heightened risks of vertical transmission following exposure to CSFV. Methods: This study evaluates the early protective efficacy of FlagT4G, a novel live attenuated DIVA-compatible vaccine. Pregnant sows were vaccinated at mid-gestation and challenged 14 days later with a highly virulent CSFV strain. Results: FlagT4G conferred complete clinical protection, preventing both maternal viremia and transplacental transmission. No CSFV RNA, specific antibodies, or IFN-α were detected in fetal samples from vaccinated animals. In contrast, unvaccinated sows exhibited clinical signs, high viral loads, and widespread fetal infection. Interestingly, early protection was observed even in the absence of strong humoral responses in some vaccinated sows, suggesting a potential role for innate or T-cell-mediated immunity in conferring rapid protection. Conclusions: The demonstrated efficacy of FlagT4G within two weeks of vaccination underscores its feasibility for integration into emergency vaccination programs. Its DIVA compatibility and ability to induce early fetal protection against highly virulent CSFV strains position it as a promising tool for CSF control and eradication strategies.

## 1. Introduction

Classical swine fever (CSF) is a highly contagious disease deemed notifiable by the World Organization for Animal Health (WOAH) with great economic importance, affecting both domestic pigs and wild boars [1,2]. Classical swine fever virus (CSFV), the etiological agent of the disease, is a member of the genus Pestivirus within the family Flaviviridae [3,4]. A major epidemiological concern associated with CSFV is its ability to cross the placental barrier, causing fetal death, congenital anomalies, or the birth of persistently infected piglets. These persistently infected animals act as immunotolerant viral reservoirs, facilitating sustained viral circulation and hampering control and eradication strategies [2,5].

Vaccination remains a pivotal tool in the control and eradication of classical swine fever (CSF), particularly in endemic regions, where it is routinely implemented as part of national programs [6,7]. Although prophylactic vaccination is prohibited in CSF-free countries except under emergency conditions, the continued circulation of CSFV in certain regions has necessitated the use of highly efficacious vaccines [8]. Conventional live attenuated vaccines have been widely adopted due to their ability to induce rapid, robust, and long-lasting immune responses [9,10]. However, a critical limitation of these vaccines is their inability to differentiate infected from vaccinated animals (DIVA), thereby restricting their application in surveillance and eradication programs, as well as in emergency vaccination strategies within CSF-free zones. In response to these limitations, significant efforts have been directed toward the development of DIVA-compatible vaccines, including subunit and chimeric approaches targeting the CSFV E2 glycoprotein [7,11].

Among the E2 subunit DIVA vaccines, Porcilis^®^ Pesti has been approved by the European Medicines Agency (EMA), although concerns remain regarding its insufficient efficacy in preventing the transplacental transmission of CSFV [12,13]. Porvac^®^, a second-generation E2 subunit vaccine incorporating the immunostimulatory molecule CD154, offers early protection against highly virulent strains with a single dose [14,15,16]; however, it still requires a booster to prevent vertical transmission, highlighting the need for improved formulations, particularly for use in pregnant sows [17]. Chimeric vaccines have emerged as a promising alternative, offering the combined benefits of live attenuated efficacy and DIVA capabilities [18,19]. The Suvaxyn^®^ CSF Marker (CP_E2alf), the first live marker vaccine approved by the European Union for emergency use [8,20], represents a major advancement; nonetheless, its efficacy against transplacental transmission has only been demonstrated under limited conditions—specifically, with moderately virulent strains and at 21 days post-vaccination [21]. Hence, an urgent demand persists for the development of CSF vaccines capable of eliciting rapid and robust immune protection against the full spectrum of clinical presentations, including acute, chronic, and persistent infections, across diverse epidemiological settings, while concurrently facilitating serological surveillance and supporting global eradication initiatives.

FlagT4G, a live attenuated vaccine candidate engineered from the CSFV Brescia strain, incorporates two antigenic markers: a FLAG epitope insertion in the E1 gene, as well as the modification of a short stretch of amino acid residues in the E2 gene, which deletes an epitope that is well conserved across CSFV strain genes [22]. Previous studies have established that FlagT4G elicits a robust immune response characterized by high immunogenicity and protective efficacy, conferring robust protection as early as three days following a single-dose administration in pigs. Notably, this vaccine also allows for reliable serological differentiation between infected and vaccinated animals (DIVA capacity), facilitating improved disease surveillance [22,23]. More recently, FlagT4G has demonstrated efficacy in preventing the vertical transmission of a highly virulent CSFV strain across the placenta in pregnant sows when administered 21 days prior to challenge, further underscoring its potential for comprehensive CSF control [24].

In endemic and emergency contexts, pregnant sows may be exposed to CSFV shortly after vaccination. This critical window of susceptibility exposes a major limitation of current CSF vaccines [2,6]. In this study, we address this critical knowledge gap by evaluating the early protective efficacy of the DIVA FlagT4G vaccine in pregnant sows challenged with a virulent CSFV strain within the first two weeks post-vaccination. We comprehensively assess clinical outcomes, viral replication, and the vaccine’s ability to prevent transplacental transmission, thereby determining its effectiveness in protecting both sows and fetuses under conditions of high-risk exposure. These results aim to expand the current evidence base supporting the implementation of DIVA vaccines in CSF eradication programs and to advance our understanding of their potential to interrupt viral persistence in swine populations.

## 2. Materials and Methods

### 2.1. Cells and Viruses

For viral production, titration, and neutralization assays, the porcine kidney cell line PK-15 (ATCC CCL-33) was used. Cells were cultured in Eagle’s Minimal Essential Medi-um (EMEM) supplemented with 5% fetal bovine serum (FBS) and incubated at 37 °C in a 5% CO_2_ atmosphere. The two viruses used in the animal study were the recombinant vac-cine candidate FlagT4G [22] and the CSFV virulent field strain Margarita, which belong to subgenotype 1.4 [24,25]. Viral titrations were carried out by end-point dilution using a peroxidase-linked assay (PLA) [26] and the titers calculated according to Reed and Muench [27]. Two CSFV strains, Diepholz and Alfort/187, were used in the neutralization assays, generously provided by the CSFV European Union Reference Laboratory (EURL) in Hanover, Germany.

### 2.2. Experimental Design

In this study, six Pestivirus-free Landrace sows at 34 days of gestation were housed under biosafety level 3 (ABSL-3) containment conditions at the IRTA-CReSA facilities (Barcelona, Spain) for the experimental procedures. The sows were randomly distributed in two groups: a vaccinated group (*n* = 4; sows 1–4) and a non-vaccinated control group (*n* = 2; sows 5–6). Following a one-week acclimatization period, at 44 days of gestation, sows in the vaccinated group received an intramuscular dose of 10^5^ TCID_50_/mL of the FlagT4G vaccine in accordance with the WOAH Manual [24]. Animals in the control group did not receive any immunization. Fourteen days after vaccination, corresponding to day 58 of gestation, both groups were challenged via intramuscular injection in the neck with 10^5^ TCID_50_/mL of the highly virulent CSFV Margarita strain. Post-vaccination and post-challenge monitoring included daily assessment of the rectal temperature and clinical signs by a trained veterinarian, using a previously established scoring system [17].

Serum, nasal, and rectal swab samples were obtained from vaccinated sows on the day of vaccination and at 6 days post-vaccination (dpv). In addition, samples were collected from all six pregnant sows on the CSFV challenge day, as well as 8 and 14 days post-challenge (dpc). At the end of the trial (14 dpc), on the 72nd day of gestation (end of the second trimester), sows were euthanized in compliance with established procedures as stipulated by European Directive 2010/63/EU. Following necropsy, tissue samples were collected from the tonsil, Peyer’s patches, and mesenteric lymph nodes.

Moreover, following the procedures previously established [17] to minimize the risk of fetal distress, the uterus was removed from the carcass and fetuses were individually retrieved following umbilical cord clamping. Fetuses were numbered sequentially: odd numbers assigned to those located in the left uterine horn and even numbers to those in the right horn, proceeding from the ovarian to the cervical end. Umbilical cord blood was obtained from each fetus for serum, and the crown-to-rump length was measured. All fetuses underwent necropsy, and fresh samples of the thymus, spleen, and tonsil were collected for further analysis. The study protocol received ethical approval from the Animal Experimentation Committee of the Generalitat of Catalonia (authorization number 10907), in accordance with current Spanish and European regulations governing animal experimentation.

### 2.3. Evaluation of Humoral Response Against CSFV E2 and Neutralizing Antibodies

A commercial ELISA kit (IDEXX Laboratories, Liebefeld, Switzerland) was employed to detect E2-specific antibodies in serum samples. The blocking percentage values were interpreted according to the manufacturer’s guidelines: values below 30% were defined as negative, values between 30% and 40% were considered as doubtful, and values exceeding 40% as positive. Additionally, neutralization peroxidase-linked antibody (NPLA) assays were conducted on the serum samples to assess neutralizing antibody titers against a panel of CSFV strains: Margarita, FlagT4G, Alfort/187, and Diepholz [28]. Neutralization titers were expressed as the reciprocal dilution of serum that neutralized 100 TCID_50_ in 50% of the culture replicates [29].

### 2.4. ELISA for IFN-α Detection in Serum Samples

To quantify the IFN-α levels in serum samples, an in-house ELISA previously described in the literature [30,31] was employed. Monoclonal antibodies against IFN-α (clones K9 and K17) and recombinant IFN-α protein (PBL Biomedical Laboratories, Piscataway, NJ, USA) were employed in the assays. Serum samples from fetuses in both experimental groups were analyzed. Cytokine concentrations (units/mL) were calculated based on a standard curve generated from the optical densities of known cytokine standards.

### 2.5. Detection of CSFV RNA

Total RNA purification from the collected samples was performed utilizing the MagAttract 96 Cador Pathogen Kit (Qiagen, Hilden, Germany), following the protocol provided by the manufacturer. Each extraction was performed on 200 μL of sample, yielding a final RNA eluate of 100 μL, stored at −80 °C. Tissues were processed by homogenization in Eagle’s Minimum Essential Medium (900 μL) supplemented with 2% antibiotic solution consisting of penicillin and streptomycin at final concentrations of 10,000 U/mL each. The supernatant, obtained after centrifugation at 13,000 rpm for 10 min, was utilized for RNA isolation. Two RT-qPCR assays were performed on all extracted RNA samples: one targeting conserved regions of CSFV RNA [32] and the other for the specific CSFV Margarita strain [33]. Amplifications were conducted using the AgPath-ID™ One-Step RT-PCR Reagents (Applied Biosystems, Waltham, MA, USA) on an ABI 7500 Real-Time PCR System. Samples were defined as positive if the cycle threshold (Ct) value was ≤40, while samples showing no detectable fluorescence were interpreted as negative. Viral RNA loads were considered as high (Ct < 23), moderate (Ct 23–28), or low (Ct 29–40). To differentiate and detect CSFV RNA from the FlagT4G and Margarita strains in the tonsils of vaccinated and unvaccinated animals, an RT-qPCR assay targeting the CSFV E2 genomic region was conducted [34], followed by Sanger sequencing with BigDye Terminator chemistry on an ABI 3130xl Genetic Analyzer (Applied Biosystems, Foster City, CA, USA). Sequence assembly and analysis were performed using the BioEdit software (v7.2) [35].

## 3. Results

### 3.1. FlagT4G Provides Clinical Protection Against CSFV Challenge in Pregnant Sows at 14 Days After Single Vaccination

**Figure 1:**
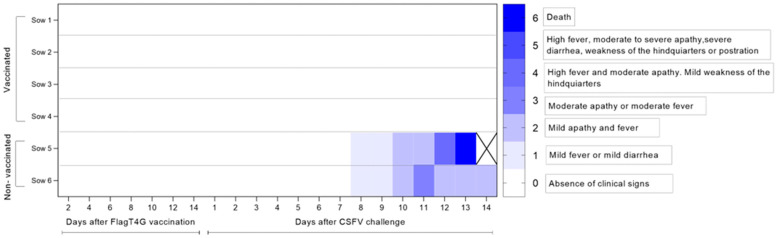
Clinical scores of pregnant sows following FlagT4G vaccination and CSFV challenge. Clinical signs were monitored daily throughout the trial. The intensity of the blue shading indicates the severity of clinical signs, with darker blue representing higher clinical scores, as defined in the legend. Sows 1 to 4 received the FlagT4G vaccine and remained clinically healthy. The control group (sows 5 and 6) showed progressive clinical deterioration after CSFV challenge. Sow 5 was euthanized on day 13 post-challenge for animal welfare reasons.

Despite undergoing CSFV challenge post-vaccination, all pregnant sows sustained a clinically healthy condition during the entire study, showing no signs of CSF. In contrast, from 8 dpc onwards, unvaccinated sows developed mild-to-moderate apathy, fever, anorexia, and mild dyspnea with nasal discharge. At 13 dpc, one non-vaccinated sow (number 5) also displayed severe apathy and prostration and was euthanized for animal welfare reasons (Figure 1). At the end of the trial, 14 days after challenge, the sows (72 days of gestation) were euthanized and a complete necropsy was performed. In non-vaccinated animals, the pre-dominant macroscopic lesions included petechial hemorrhages in the kidneys, tonsils, and spleen—characteristic findings of CSF. In contrast, macroscopic pathological lesions were absent in all vaccinated sows (Figure 1).

### 3.2. Robust Humoral Immune Response Evidenced by E2-Specific and Neutralizing Antibody Production in FlagT4G-Vaccinated Pregnant Sows

**Figure 2:**
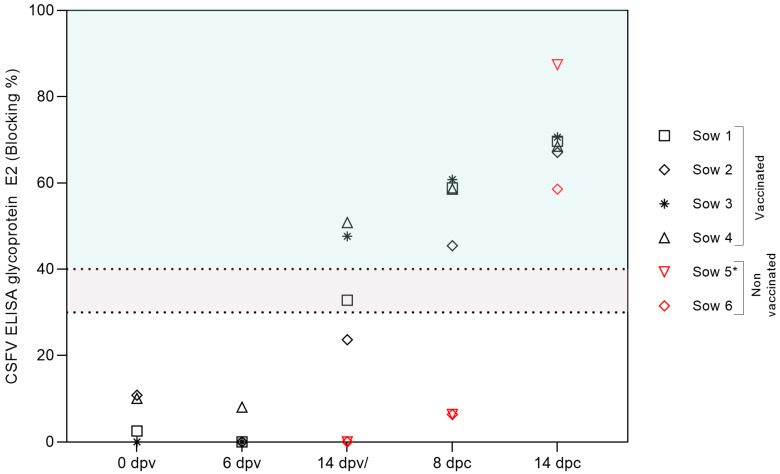
Humoral immune response generated in pregnant sows after FlagT4G vaccination and CSFV challenge. Detection of E2-specific antibodies in sera from FlagT4G-immunized (black symbols) and non-immunized (red symbols) pregnant sows using a commercial ELISA test. Data are presented as percentages of inhibition. Inhibition values between 30% and 40% fall within the doubtful range (grey-shaded area), while values exceeding 40% are considered positive (green-shaded area). * = sample taken at 13 dpc.

**Figure 3:**
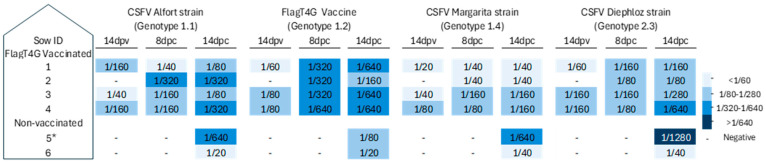
Kinetics of neutralizing antibody response in FlagT4G-vaccinated and non-vaccinated sows. Neutralizing antibody titters following FlagT4G vaccination and CSFV challenge were assessed weekly using the NPLA assay. Antibody titers, ranging from low to high, are shown using a color scale from light to dark blue. Negative samples are denoted by the symbol “–”. * = sample taken at 13 dpc.

The generation of CSFV-specific antibodies following vaccination and subsequent exposure to a highly virulent strain of CSFV was analyzed in the collected serum samples (Figure 2). By 14 dpv, two of the four vaccinated sows (numbers 3 and 4) showed detectable levels of antibodies against the CSFV E2 glycoprotein, with blocking percentages of 47 and 50, respectively. By 8 days post-challenge (dpc), all four vaccinated sows showed strong seroconversion, with blocking values around 70%, as determined by a commercial CSFV E2 glycoprotein-specific antibody ELISA. The development of neutralizing antibodies against CSFV was also confirmed via serum neutralization testing, which revealed a rapid increase in antibody titers in all vaccinated animals following challenge by 8 dpc (Figure 3). Notably, sow number 1, which tested negative by ELISA at 14 dpv, exhibited neutralizing antibody titers of 1:60 against different CSFV strains representative of genotypes 1 and 2. In the vaccinated group, a substantial rise in antibody titers was observed at 8 and 14 dpc, reaching up to 1:640. In contrast, in the unvaccinated group, the detection of E2-specific antibodies occurred only at the final sampling points: 13 dpc in sow 5 and 14 dpc in sow 6 (Figure 3). Additionally, sow number 6 displayed low neutralizing antibody titers, ranging from 1:20 to 1:40, whereas sow number 5 showed significantly higher titers, reaching up to 1:1280 (Figure 3).

### 3.3. Absence of Viremia and Viral Shedding in Pregnant Sows Two Weeks After Single-Dose FlagT4G Vaccination

**Figure 4:**
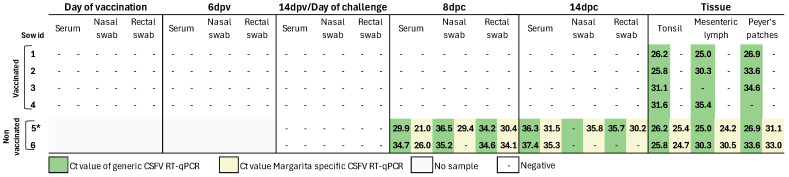
CSFV RNA detection in pregnant sows following FlagT4G vaccination and subsequent challenge. Viral RNA was detected using RT-qPCR in serum, nasal swabs, and rectal swabs collected weekly, as well as in tissues obtained during post-mortem examination. The CSFV Margarita strain-specific RT-qPCR is represented in pink, while the CSFV generic RT-qPCR is shown in blue. Negative samples are indicated by the symbol “–”. * = sample taken at 13 dpc.

In all samples obtained from vaccinated sows, either post-vaccination or post-challenge with the highly virulent CSFV strain, no RNA corresponding to the CSFV Margarita strain was detected using the strain-specific RT-qPCR assay (Figure 4). Notably, serum, nasal, and rectal swabs from vaccinated animals tested negative for FlagT4G vaccine-derived RNA at all time points after vaccination and challenge, as determined by the generic CSFV RT-qPCR assay (Figure 4). However, tonsil samples from all vaccinated sows tested positive by the generic CSFV RT-qPCR assay, with cycle threshold (Ct) values ranging from 25.9 to 31.6, indicative of moderate-to-low levels of FlagT4G vaccine RNA. Furthermore, mesenteric lymph nodes and Peyer’s patches tested positive in three out of four immunized sows, as evidenced by Ct values indicative of moderate-to-low viral RNA loads (Ct range: 25–35). In contrast, samples from unvaccinated, challenged sows revealed widespread viral presence. At 8 dpc, both the generic and Margarita strain-specific RT-qPCR assays detected CSFV RNA in all serum, nasal, and rectal swab samples (Figure 4), yielding Ct values indicative of low-to-moderate RNA viral loads. By 14 dpc, samples from sow number 5 remained positive in both assays except for the nasal swab, in which case only the Margarita strain-specific assay detected viral RNA. In sow number 6, CSFV RNA was found only in the serum by the CSFV Margarita strain-specific RT-qPCR. Moreover, both RT-qPCR assays detected CSFV RNA in the mesenteric lymph nodes, Peyer’s patches, and tonsils of unvaccinated sows, with Ct values indicative of moderate-to-high viral RNA loads (Ct range: 25–33) (Figure 3).

### 3.4. Complete Early Protection of Fetuses Against Highly Virulent CSFV Challenge Following Single-Dose FlagT4G Vaccination in Pregnant Sows

**Figure 5:**
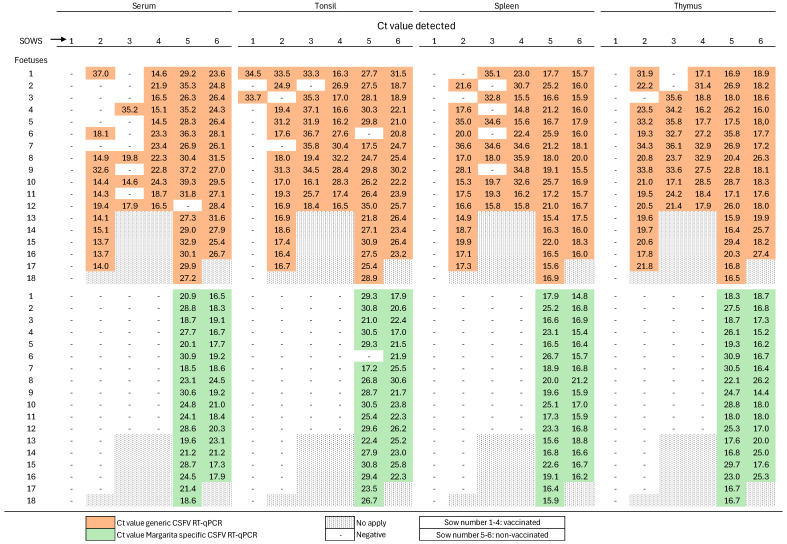
Detection of CSFV RNA in fetuses of vaccinated and unvaccinated sows following CSFV challenge using RT-qPCR. Detection of CSFV in fetal samples was carried out using two RT-qPCR assays. Data from the CSFV Margarita strain-specific RT-qPCR are visualized in green, and those from the generic RT-qPCR assay are visualized in orange. The symbol “–” indicates a negative result. “No apply” indicates that the sow did not gestate fetuses in the given position.

After necropsy was performed at 72 days of gestation (28 dpv, 14 dpc), in fetuses from FlagT4G-vaccinated sows, no CSFV RNA was detected in the serum or any collected tissues (spleen, tonsil, or thymus) by the Margarita strain-specific RT-qPCR assay (Figure 5). Notably, using the generic CSFV RT-qPCR assay, CSFV RNA was detected in the thymus, tonsil, and spleen samples of three of the four vaccinated and challenged sows (sows 2, 3, and 4), with Ct values ranging from 14 to 36, thus indicating variable levels of FlagT4G vaccine RNA. Notably, only two fetal tonsil samples from sow 1 tested positive by the generic RT-qPCR assay (Figure 5). To confirm the identity of the detected viral RNA, RT-PCR targeting the E2 gene was performed on fetal tonsil samples, followed by sequencing. Only FlagT4G vaccine RNA was identified in these samples, with no detection of wild-type CSFV, thereby confirming the lack of transplacental transmission of the challenge virus in vaccinated groups.

Conversely, fetuses from unvaccinated sows exhibited substantial variability in size and presented with multiple gross pathological lesions, including hemorrhages in the skin, intestines, kidneys, and spleen. Molecular analysis of fetus samples using the Margarita strain-specific RT-qPCR assay confirmed the presence of CSFV RNA in serum, thymus, tonsil, and spleen samples. Ct values ranged from 14 to 30, consistent with moderate-to-high RNA loads across most tissues analyzed (Figure 5). The presence of infectious virus was further validated through successful viral isolation from samples with Ct values below 30.

### 3.5. Lack of Humoral and Type I Interferon Responses in Fetuses from FlagT4G-Vaccinated Sows Following CSFV Challenge

**Figure 6:**
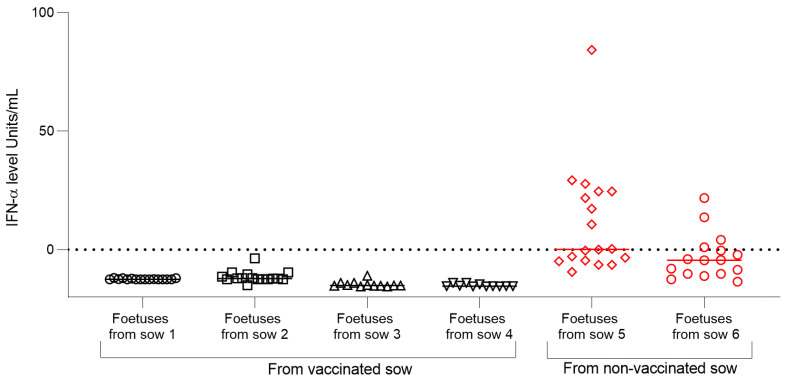
Comparison of fetal IFN-α responses between vaccinated and unvaccinated sows after CSFV challenge. Concentrations of the cytokine IFN-α were determined in serum samples collected from fetuses of vaccinated (black symbols) and non-vaccinated (red symbols) sows following CSFV challenge, using an in-house ELISA assay. Individual values are expressed in units per milliliter (U/mL).

No specific antibodies against the CSFV E2 glycoprotein were detected in fetuses from any experimental group throughout the study period. Regarding the innate immune response, several fetuses from unvaccinated sows exhibited increased levels of interferon-alpha (IFN-α), with concentrations ranging from 20 to 85 units/mL (Figure 6). In contrast, the IFN-α concentrations in fetuses from vaccinated-CSFV-challenged sows remained undetectable, suggesting the absence of viral infection or systemic immune activation in these animals (Figure 6).

## 4. Discussion

Despite the official recognition of a CSF-free status in 37 countries, CSF remains a significant global threat due to persistence in endemic regions and resultant restrictions on pig movement and trade [36,37,38]. Live marker vaccines are pivotal in CSF control programs, as they elicit strong immunity and support surveillance through DIVA strategies [7,39]. However, the early protective capacity of the currently available live marker vaccines against the transplacental transmission of CSFV within two weeks of vaccination is not yet known—a critical issue in endemic and emergency settings, where pregnant sows may be exposed shortly after immunization [2,5,9]. Our study offers the first evidence that the FlagT4G marker vaccine confers complete early protection against highly virulent CSFV challenge as early as 14 days following vaccination in pregnant sows. The viral challenge was performed in mid-gestation, a period prone to fetal death, congenital anomalies, or persistent infection when exposed to CSFV [2,5]. FlagT4G safely prevented fetal infection; vaccinated sows and fetuses exhibited no CSF clinical signs; and the challenge strain remained undetectable in all tissues. Importantly, no CSFV-specific antibodies or IFN-α were detected in fetal sera, indicative of the absence of both adaptive and innate immune responses to the challenge virus, thus complying with established regulations on vaccine safety in fetuses [36].

At the time of challenge, only 50% of the vaccinated sows displayed low titers of CSFV-neutralizing antibodies. Nevertheless, upon viral exposure, all vaccinated animals mounted a rapid and robust anamnestic antibody response, characterized by high titers of neutralizing antibodies against both genotypes 1 and 2. This vigorous neutralizing activity was closely associated with complete clinical protection, even under the stringent challenge conditions stipulated by the WOAH Manual and applied in the present study. These findings are consistent with previous reports describing the early induction of neutralizing antibodies as early as 4 days post-challenge in FlagT4G-vaccinated pigs, despite the absence of detectable pre-challenge antibody levels. These results align with prior observations of early neutralizing antibody emergence as soon as 4 days post-challenge in FlagT4G-vaccinated pigs, despite pre-challenge seronegativity [40], and they complement evidence from CSFV-based vaccines in pigs and pregnant sows. Our findings further highlight the indispensable role of the humoral immune response in CSFV protection and suggest the presence of low-frequency memory B-cells capable of rapid reactivation upon antigen re-encounter—a mechanism that is well documented in both murine and human models of secondary immunity. Indeed, investigations into CSFV support this hypothesis, demonstrating memory B-cell-mediated recall responses even in the absence of detectable antibodies at the time of CSFV challenge [41]. To further substantiate these observations, future studies are essential. Such work should aim to delineate the kinetics, specificity, and anatomical distribution of memory B-cell populations, employing tools like ELISPOT and flow cytometry, as utilized in SARS-CoV-2 immunology [42], in large animal models, including pregnant sows. This will be critical to validating the hypothesized memory B-cell contribution to rapid secondary humoral responses.

In contrast, unvaccinated sows developed pronounced clinical disease, sustained viremia, and elevated viral loads, thereby further substantiating the protective efficacy of the FlagT4G vaccine candidate. The detection of vaccine-derived viral RNA, without the concurrent identification of the challenge strain, in selected fetal tissues raises critical considerations regarding the kinetics and safety of the transplacental transmission of live attenuated vaccine viruses. Nevertheless, it is important highlight that a high vaccine dose was administered in this study, which may have influenced the extent of fetal exposure. Recent studies have demonstrated the genetic stability, favorable safety profile, and strong protective immunogenicity of FlagT4G in fattening piglets, even at doses as low as 10^2^ TCID_50_ [24,43]. This dose is proposed as the starting point for future investigations in pregnant sows to further evaluate vaccine safety and the dynamics of potential vertical transmission.

## 5. Conclusions

This study provides the first evidence that the FlagT4G live attenuated marker vaccine confers complete clinical and virological protection against a highly virulent CSFV challenge in pregnant sows as early as 14 days post-vaccination. Vaccinated animals displayed no clinical signs, viremia, or detectable challenge virus in either maternal or fetal tissues. Furthermore, the absence of fetal immune activation underscores the vaccine’s safety during mid-gestation, a critical window of heightened fetal susceptibility. Strikingly, despite low or undetectable antibody levels at the time of challenge, all vaccinated sows mounted a rapid and robust anamnestic antibody response, underscoring the pivotal involvement of memory B-cells. These findings also highlight the indispensable role of neutralizing antibody-mediated responses in achieving protection against CSFV, thereby reinforcing the central contribution of humoral immunity to disease control. Taken together, these results position FlagT4G as a promising candidate for the effective immunization of pregnant sows within both CSF-endemic regions and emergency vaccination programs.

## Data Availability

The original contributions presented in the study are provided within the manuscript.
